# Association of erectile dysfunction with coronary artery disease in Type 2 Diabetes mellitus

**DOI:** 10.4314/gmj.v57i1.7

**Published:** 2023-01

**Authors:** Sydney C Dsouza, Obaid Rahman

**Affiliations:** Department of General Medicine, Yenepoya Medical College, Mangalore, India

**Keywords:** Erectile dysfunction, Diabetes Mellitus, Coronary angiogram, coronary artery disease, chronic disease

## Abstract

**Objectives:**

Association of severity of Erectile Dysfunction (ED) and coronary artery disease (CAD) in type 2 diabetics based on the number of vessels involved

**Design:**

an observational, cross-sectional study

**Setting:**

tertiary level health care centre

**Participants:**

104 diabetics, as defined by ADA(American Diabetes Association) criteria, who fulfilled the inclusion criteria of positive coronary angiogram (in the last six months), were selected to participate in the study after obtaining informed consent. Details regarding ED were obtained using the IIEF-5 questionnaire, and based on their scores, participants were divided into four categories ranging from mild to severe.

**Interventions:**

use of questionnaire International Index of Erectile Function-5

**Main outcome measures:**

Karl Pearson association was done between the number of major coronary vessels involved and the severity of ED. The receiver operating characteristic curve was plotted between ED status and coronary vessels involved to predict the cut-off limit of ED score to predict CAD.

**Results:**

Out of 104 diabetics with CAD, 85.5% gave a history of ED. Most participants had mild to moderate degrees of ED, which was reported as occurring 4-6 years before the diagnosis of CAD. Cross tabulation between the severity of ED and association with the number of coronary vessels involved found no statistically significant association (p>0.05). However, ROC analysis showed sufficient accuracy in predicting the severity of CAD.

**Conclusion:**

The presence of ED in diabetic patients warrants screening for cardiovascular disease. A clear association between the severity of ED and the number of coronary vessels involved is still questionable.

**Funding:**

None declared

## Introduction

Diabetes Mellitus is a non-communicable, chronic, metabolic disease characterised by elevated blood glucose levels and, over time, may be associated with serious organ complications. According to the International Diabetes Federation,^1^ about 536 million adults between the age of 20 to 79 years are living with diabetes; by 2045, this is expected to rise to 700 million. India ranks second highest in the world, with an estimated 73 million diabetes cases in 2021.^2^

Diabetes mellitus leads to the development of both micro and macrovascular complications. Erectile dysfunction is a macrovascular (though other mechanisms also might play a role) complication, and this has been gaining importance because, all over the world, 35 to 75 % of diabetic men (about 150 million) are affected.^3^ Erectile dysfunction (ED) is an inability to generate and sustain a penile erection for sexual intercourse.

Both psychological and organic factors can contribute to ED. The proposed mechanisms of ED in diabetic patients are vasculopathy, neuropathy, visceral adiposity, insulin resistance and hypogonadism.^4^ Decreased nitric oxide availability in a diabetic patient can lead to endothelial dysfunction. This results in improper relaxation of the vascular smooth muscle of corpora cavernosa. To check the status and the severity of erectile dysfunction, the International Index of Erectile Function Questionnaire (IIEF-5) has been used.^5^

Coronary artery disease (CAD) is an established cause of morbidity and mortality in the diabetic population. The gold standard test to detect coronary artery involvement is a coronary angiogram. More than 50% of a single major artery involved is considered significant CAD.^6^

The link between ED and cardiovascular disease is mostly due to the sustained hyperglycaemia on the endothelium, which results in endothelial dysfunction. Long-standing hyperglycaemia produces accelerated atherosclerosis, eventually leading to increased mortality and morbidity.^7^ The Global Burden of Disease (GBD) study approximates the age-standardised CVD (cardiovascular disease) death rate of 272 per 100000 population in India, which is higher than the world (235 per 100000).^8^

The CVD epidemic in India is a cause for concern due to the early onset of the disease condition and increased case fatality rate.^8^ Nearly a quarter of the deaths in India is due to CVD. 8 Despite overall development in health technology, healthcare access remains a troublesome factor. Many patients do not have ready access to interventional cardiac care, adding to the morbidity and mortality. If a non-invasive, simple tool like the IIEF-5 can help identify diabetics at risk for CAD, this will go a long way in reducing the burden on the healthcare system. Hence, we saw a need to study the association between the severity of ED and the number of coronary arteries involved in the Indian diabetic population. Our study was conducted to analyse the association of severity of ED and CAD in patients with diabetes based on the number of vessels involved. Though there are few studies describing this association, by and large, this problem has been understudied in our country.

## Methods

A cross-sectional study was conducted in the outpatient and inpatient department of a tertiary care teaching hospital in South India over one year. After obtaining approval from the Institutional Ethics Committee, a study population of 104 Type 2 diabetic males who fulfilled the ADA (American Diabetes Association) criteria were enrolled in the study. All 104 participants had undergone cardiac catheterisation and had a positive coronary angiogram (CAG) report (minimum of 50% blockage in at least one major coronary artery) within the last six months. Patients with a history of depression, trauma, smoking, Peyronie`s disease, neurological disorder, radical prostatectomy, taking drugs liable to produce erectile dysfunction, other endocrine disorder and patients with ED who were on treatment were excluded. A signed informed consent form was obtained from each participant after counselling and reading out the participant information sheet. The questionnaire on ED - International Index of Erectile Function (IIEF-5) was administered, taking care of privacy and confidentiality.

Questions were either self-administered in patients who were literate or with the help of the researcher.

The International Index of Erectile Function (IIEF-5) questionnaire is an accepted tool to assess sexual function. It consists of five components of sexual function such as orgasm, erectile function, desire for sex, post-intercourse and overall satisfaction. Each aspect is measured on a five-point scale, and a score of > 21 is considered normal erectile function. Based on the scores, our participants were further classified into mild (17–21), mild to moderate (12–16), moderate (8–11) and severe ED (less than 8).^5^

### Statistical Analysis

Karl Pearson association was made between the number of major coronary vessels involved and the severity of erectile dysfunction. SPSS version number 23 (IBM, Chicago, USA) with 5% significance level. The Receiver Operating Characteristic curve was plotted between ED status and coronary vessels involved to predict the cut-off limit of ED score to predict CAD.

## Results

The study included one hundred and four patients with a mean age of 55 ± 8.12 years. The demographic details of the population and data analysed are summarised in Table 1.

**Table 1:** Demographics of Population Studied

Demographics		Frequency N(%)
**Age (years)**	36–45	17(16.34)
	46–55	35(33.65)
	56–65	42(40.38)
	>65	10(9.61)
**Erectile Dysfunction**	Yes	89(85.6)
	No	15(14.4)
**Duration of ED** **(years)**	No ED	15(14.4)
	0–3	23(22.1)
	4–6	37(35.6)
	7–9	11(10.6)
	>10	18(17.3)
**Severity of ED**	No ED	15(14.4)
	Mild	18(17.3)
	Mild to moderate	44(42.3)
	Moderate	20(19.2)
	Severe	7(6.7)
**Coronary artery disease**		
	Single vessel	26(25.2)
	Double vessel	44(41.7)
	Triple vessel	34(33)

**Figure 1:**
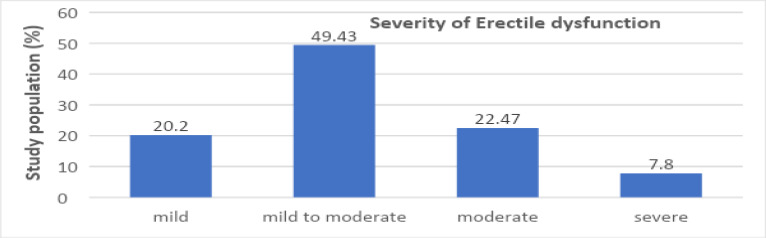
Bar diagram showing the distribution of severity of ED

The majority of the study population had ED (85.6%). The severity of ED was further classified into mild, moderate and severe; a large number of patients (49.43%) had mild to moderate ED, as shown in Figure 1. Among the study population, 26 (25.96%) had single-vessel disease (SVD), 44 (42.3%) had double vessel disease (DVD), and 34 (32.7%) had triple vessel disease (TVD). When comparing the number of coronary arteries involved with the presence of ED, the severity of ED did not worsen with an increase in the number of coronaries involved (p>0.05).

About forty per cent (35.9% of the participants had 4 - 6 years of ED; among them, an equal number had DVD and TVD involved (13.6%, 13.6%). There was an association between the participant's age and the duration of erectile dysfunction. As age advanced, the duration of ED increased. (p<0.05) as revealed in Table 3.

**Table 3:** Age of the study population with a duration of erectile dysfunction

Age of the study population	Duration of Erectile dysfunction					Total N (%)
	No ED N (%)	0–3 years N (%)	4–6 years N (%)	7–9 years N (%)	>10 years N (%)	
**36–45**	10 (9.6%)	1 (1.0%)	5 (4.8%)	1 (1.0%)	0 (0.0%)	17 (16.3%)
**46–55**	5 (4.8%)	17 (16.3%)	12 (11.5%)	0 (0.0%)	1 (1.0%)	35 (33.7%)
**56–65**	0 (0.0%)	5 (4.8%)	20 (19.2%)	7 (6.7%)	10 (9.6%)	42 (40.4%)
**>65**	0 (0.0%)	0 (0.0%)	0 (0.0%)	3 (2.9%)	7 (6.7%)	10 (9.6%)
**Total**	15 (14.4%)	23 (22.1%)	37 (35.6%)	11 (10.6%)	18 (17.3%)	104 (100%)

There was an association between the older age group and the severity of erectile dysfunction. (P<0.05). As the age advanced severity of ED increased, as evidenced by a p-value <0.05. (Table 4).

**Table 4:** Age of the sample population and severity of ED

Age of the sample population	Severity of Erectile dysfunction					Total N (%)
	No ED N (%)	Mild N (%)	Mild to moderate N (%)	Moderate N (%)	Severe N (%)	
**36–45**	10 (9.6%)	1 (1.0%)	5 (4.8%)	1 (1.0%)	0 (0.0%)	17 (16.3%)
**46–55**	5 (4.8%)	11 (10.6%)	15 (14.4%)	4 (3.8%)	0 (0.0%)	35 (33.7%)
**56–65**	0 (0.0%)	4 (3.8%)	22 (21.2%)	13 (12.5%)	3 (2.9%)	42 (40.4%)
**>65**	0 (0.0%)	2 (1.9%)	2 (1.9%)	2 (1.9%)	4 (3.8%)	10 (9.6%)
**Total**	15 (14.4%)	18 (17.3%)	44 (42.3%)	20 (19.2%)	7 (6.7%)	104 (100%)

The ROC shows that ED scores have sufficient accuracy in predicting the severity of CAD (DVDs/TVDs) in diabetic patients, as the area under the ROC (AUC) is 0.624 (>0.5). The cut-off limit for the same is obtained by considering the coordinates that give an ED score of **15** here.

## Discussion

ED diagnosed using the IIEF-5 questionnaire was quite common among our diabetic participants (85.6%) and preceded the diagnosis of CAD on an average of 4-6 years before the angiography was performed. Most participants (49.43%) had mild to moderately severe ED. In our study, the severity and duration of erectile dysfunction had a significant association with age. The severity and duration of erectile dysfunction increased with age. The association between the severity of ED and the number of coronaries involved was statistically insignificant. However, ROC analysis between ED status and coronary artery disease status shows that ED scores have sufficient accuracy in predicting the severity of CAD (DVDs/TVDs) in diabetic patients, with the area under the ROC (AUR) being 0.624 (>0.5).

ED is a growing health concern that has notably impacted the quality of life, especially among diabetic males. Despite being a preventable diabetic complication, the prevalence of ED ranges from 35 to 90% and a 2-3 times higher occurrence in men with diabetes than those without diabetes.7 Due to the taboo on sexuality, this aspect of ED is largely suppressed by the patients and not given due importance by the medical personnel.

ED is now identified as a generalised vascular disorder with abnormal physiologic and organic factors affecting the penile circulation^9^. Multiple interrelated factors like psychogenic, neurogenic, hormonal, drug-induced, life-style, systemic disorders and vasculogenic are involved in the development of ED. Vasculogenic ED has gained importance in the recent decade.^9^ Conditions such as priapism, trauma, radiation therapy, surgery and veno-occlusive disorders contribute to the above. Chronic hypoxia, atherosclerotic risk factors and arterial insufficiency also can lead to reduced smooth muscle relaxation of the corpora cavernosa by reduced neuronal or endothelial nitric oxide and tissue ischaemia leading to ED.^9^

Endothelial dysfunction plays a major role in the pathogenesis of ED^10^. An essential pathophysiological connection to vasculogenic ED is atherosclerosis, diabetes mellitus, coronary artery disease, arterial hypertension, dyslipidaemia and renal disease. As a result, it has been observed that penile arterial blood flow is compromised much earlier in male patients with systemic atherosclerosis than in coronary blood flow.^10^ Furthermore, due to the similar pathological basis of endothelial dysfunction, it has been established that both ED and cardiovascular disease (CVD) coexist, making it an early indicator for CAD in a diabetic population.^11^

Age and erectile dysfunction are strongly correlated.^12^,^13^ According to two landmark studies, the Massachusetts Male Ageing Study (MMAS),^12^ men between 40-70 years of age had a 52% incidence of ED which strongly correlated with age, health and emotional status. On the other hand, the European Male Ageing Study (EMAS) 13 reported an average of 30% incidence in the same age. Similarly, in a study done in Olmstead County,^14^ men had a 50-fold higher risk of CVD in the age group of 40-49 years.

In a study done by Nutalapati et al.,^15^ a univariate analysis of the study population showed ED was more prevalent in older patients than controls. A linear relationship between increasing ED occurrence and age has been reported in studies and has posed an essential risk factor for ED.^15^ In our present study, the severity and duration of erectile dysfunction had a significant association with age. It showed that the severity and duration of erectile dysfunction increased with the age of the participants. As age advances, the number of coronary artery involvement also increases. This appears to be following earlier studies.^12^,^13^ It implies that as age advance severity of ED also increases.

In a study involving Asian Indians by Kumar J et al.,^16^ severe CAD with multi-vessel involvement was present in patients with ED. Hence, it is essential to enquire about the sexual history of a young male with CVD risk to aid in the early diagnosis of CAD. However, in this study, 39% were diabetics, and 42% were chronic smokers.

In a similar study in a tertiary hospital in Goa17 that recruited a younger age group, subjects with ED were three times more (OR = 2.98; 1.1-3.0) likely to have at least one coronary artery involved than the non-ED group. ED was significantly higher in the diabetic group (65%) than in non-diabetics (39.4%). In our study, Pearsons`s Scoring was done between ED status associated with the number of coronary vessels involved and was found to be statistically insignificant (>0.05). In the present study, all participants were diabetic, and many had double vessel disease, which could have led to the lack of statistical significance.

Observations from a study done by Salem et al. 18 have shown that ED is a predictor of CVD in men. Poor glycaemic control and nitric oxide synthesis disorder result in increased reactive oxygen species (ROS), which causes endothelial damage. ED may be the only presentation in a diabetic with asymptomatic CAD. According to Gazzaruso et al.,^19^ ED was found in 33.8% of diabetics with silent ischaemia compared to only 4.7% without silent ischaemia. In a study conducted on elderly diabetic men with diabetes, the analysis confirmed ED as a reliable predictor of asymptomatic CAD in young diabetics compared to the elderly.^20^

ROC Analysis between ED status and coronary artery disease status shows that ED scores have sufficient accuracy in predicting the severity of CAD (DVDs/TVDs) in diabetic patients as the area under the ROC (AUR) is 0.624 (>0.5). The ED cut-off score can be taken as 15 or below for predicting the severity of CAD. A study by Feng-Juan Yao et al. 21 on erectile dysfunction stated that ED might appear even before the conventional cardiovascular risk factors and may be the earliest clinical sign of subclinical cardiovascular disease, even in young adults.

A limitation of this study was that we did not attempt to correlate the severity and duration of diabetes with the severity of ED. An early diagnosis of ED can decrease morbidity and enhance primary prevention of CVD, especially in diabetics. Individuals experiencing any symptoms of ED should undergo screening to look for CVD risk to utilise effective lifestyle/pharmacological intervention. An ED cut-off score of 15 or below can be a surrogate marker for predicting CAD in diabetics.

## Conclusion

Erectile dysfunction is a common concern in older men affected with diabetes. This study looked for an association between the severity of ED with the number of coronary vessels angiographically involved.

In our study, a significant number (85.5%) of Type 2 diabetics gave a history of ED before angiographic evidence of coronary artery involvement. However, the number of coronary arteries involved was not directly associated with the severity of ED.

## Figures and Tables

**Figure 2:**
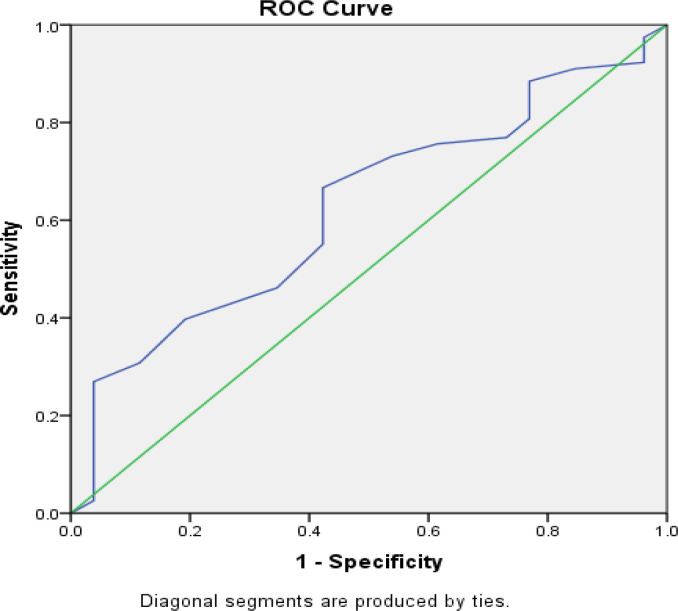
ROC for ED Score and CAD status (0.624 for predicting the cut-off limit of ED score, which is 15).

**Table 2:** Comparison between ED status and coronary artery disease

Coronary artery disease	Erectile dysfunction status					Total N (%)
	NO ED N (%)	Mild N (%)	Mild to Moderate N (%)	Moderate N (%)	Severe N (%)	
**Single vessel**	3 (2.9%)	5(4.9%)	9(8.7%)	7(6.8%)	2(1.9%)	26(25.2%)
**Double vessel**	9(8.7%)	7(6.8%)	17(16.5%)	7(6.8%)	3(2.9%)	44(41.7%)
**Triple vessel**	3(2.9%)	6(5.8%)	17(16.5%)	6(5.8%)	2(1.9%)	34(33%)
**Total**	15(14.6%)	18(17.5%)	43(41.7%)	20(19.4%)	7(6.8%)	104(100%)
